# Characterization of a Suppressive *Cis*-acting Element in the Epstein–Barr Virus LMP1 Promoter

**DOI:** 10.3389/fmicb.2017.02302

**Published:** 2017-11-22

**Authors:** Masahiro Yoshida, Takayuki Murata, Keiji Ashio, Yohei Narita, Takahiro Watanabe, H. M. Abdullah Al Masud, Yoshitaka Sato, Fumi Goshima, Hiroshi Kimura

**Affiliations:** ^1^Department of Virology, Nagoya University Graduate School of Medicine, Nagoya, Japan; ^2^Department of Virology and Parasitology, Fujita Health University School of Medicine, Toyoake, Japan

**Keywords:** EBV, LMP1, promoter, transcription, EBV-BAC

## Abstract

Latent membrane protein 1 (LMP1) is a major oncogene encoded by Epstein–Barr virus (EBV) and is essential for immortalization of B cells by the virus. Previous studies suggested that several transcription factors, such as PU.1, RBP-Jκ, NFκB, EBF1, AP-2 and STAT, are involved in LMP1 induction; however, the means by which the oncogene is negatively regulated remains unclear. Here, we introduced short mutations into the proximal LMP1 promoter that includes recognition sites for the E-box and Ikaros transcription factors in the context of EBV-bacterial artificial chromosome. Upon infection, the mutant exhibited increased LMP1 expression and EBV-mediated immortalization of B cells. However, single mutations of either the E-box or Ikaros sites had limited effects on LMP1 expression and transformation. Our results suggest that this region contains a suppressive *cis*-regulatory element, but other transcriptional repressors (apart from the E-box and Ikaros transcription factors) may remain to be discovered.

## Introduction

The Epstein–Barr virus (EBV) is a gamma-herpesvirus that infects humans. The virus principally infects B cells, establishing latent infections in such cells, but can also infect other cell types, including epithelial cells and other types of lymphocytes, such as T cells and natural killer (NK) cells. EBV infection has been implicated in infectious mononucleosis and a variety of malignancies, such as Burkitt lymphoma, NK/T cell lymphoma and nasopharyngeal carcinoma (NPC). The expression pattern of viral latent genes is dependent largely on the tissue of origin and the state of the tumor (Murata et al., 2014). EBV in Burkitt lymphoma or gastric carcinoma exhibits type I latency, in which only EBV-encoded small RNAs (EBERs) and EBV nuclear antigen 1 (EBNA1) are expressed. In type II latency (some Hodgkin lymphomas, NPC, and NK/T cell lymphoma), the genes encoding latent membrane protein 1 (LMP1) and LMP2 are expressed in addition to the EBERs and EBNA1. EBV produces EBNA2, EBNA3 and EBNA-LP, as well as EBERs, EBNA1, LMP1 and LMP2 in immunosuppression-related lymphoma or lymphoblastoid cell lines (LCLs) (type III latency). LMP1 constitutively activates cellular signaling through NFκB, MAPK, JAK/STAT, and AKT and is believed to be a major oncogene encoded by EBV (Kilger et al., 1998; Lam and Sugden, 2003; Shair et al., 2007; Kieser and Sterz, 2015).

Two promoters regulate LMP1 gene transcription. In terms of latency III infection of B cells, LMP1 is transcribed principally from the proximal ED-L1 promoter in an EBNA2-dependent manner (Kempkes and Ling, 2015). EBNA2 cannot directly bind to DNA, but it can activate a subset of promoters, including the proximal LMP1 promoter or the C promoter for EBNAs, because EBNA2 binds to certain transcription factors, such as the recombination signal binding protein Jκ (RBP-Jκ) and PU-box 1 (PU.1), and enhances the transcriptional activity by serving as a cofactor (Laux et al., 1994; Johannsen et al., 1995). In addition, two other viral proteins, EBNA-LP and EBNA3C, can interact with the EBNA2 complex and further modify the transcription (Nitsche et al., 1997; Zhao and Sample, 2000).

In contrast, LMP1 is transcribed in an EBNA2-independent manner in type II latency, during which EBNA2 is not produced. LMP1 transcription is induced by the activation of JAK/STAT signaling pathway mediated by several types of cytokines, such as IL-4, IL-6, IL-10, IL-13, and IL-21 (Chen et al., 2003; Kis et al., 2006, 2010). In the latency II situation, the STAT-regulated distal LMP1 promoter is mainly employed (Sadler and Raab-Traub, 1995; Kis et al., 2010), in addition to the proximal promoter. The distal promoter is termed the TR-L1p because it is located in the terminal repeats (TRs).

Moreover, the involvement of other transcription factors, such as NFκB (Demetriades and Mosialos, 2009; Johansson et al., 2009), AP-2 (Jansson et al., 2007; Murata et al., 2016), POU domain protein (Sjöblom et al., 1995), ATF/CREB (Sjöblom et al., 1998), SP1/3 (Tsai et al., 1999), IRF7 (Ning et al., 2003), C/EBP (Noda et al., 2011), EBF1 (Zhao et al., 2011; Lu et al., 2016; Murata et al., 2016), and CTCF (Chen et al., 2014) has been reported.

Despite these well-targeted, focused studies, most mutagenesis work has been performed *in vitro* or with the aid of reporter assays. Functional analysis of the *cis*-acting elements using point-mutated recombinant virus has not been performed yet.

The enhancer box (E-box) is a DNA element found in eukaryotes that plays roles in immunoglobulin production, myogenesis, the circadian clock, cell proliferation, and B cell development. Transcription factors, such as c-Myc, Max and Mad/Mxd, can target palindromic E-box sequence, CACGTG, and facilitate or repress transcription (Amati et al., 2001; Luscher and Vervoorts, 2012; Wahlstrom and Henriksson, 2015).

The E-box motif in the proximal LMP1 promoter is bound by various transcription factors, including Max, Mad/Mxd and TCF3/E47, but not by c-Myc, and has been suggested to play an inhibitory role in LMP1 transcription (Sjöblom-Hallén et al., 1999).

The physiological significance of such inhibition of LMP1 expression remains unknown, but lower levels of LMP1 may be beneficial in terms of the survival of infected cells or virus replication; accumulating evidence suggests that LMP1 may be toxic to cells at least under certain conditions (Lu et al., 1996; Ito et al., 2014).

Sjöblom-Hallén et al. (1999) investigated the motif using only CAT assays. Such reporter assays can be artificial and do not always reflect actual behavior *in vivo*. In addition, the cited authors used a reporter plasmid, in which a short nucleotide stretch (-67 to -55) was mutated, but the mutation unexpectedly contained a recognition motif for another transcription factor, Ikaros (Jansson et al., 2007).

Ikaros, also known as Ikaros family zinc finger protein 1 (IKZF1), has zinc-finger DNA binding motifs and is abundantly expressed in lymphocytes (Georgopoulos et al., 1994; Molnár and Georgopoulos, 1994). It binds to consensus sequence TGGGA(A/T) and regulate transcription of the target genes.

In the present study, we prepared luciferase assay vectors and recombinant EBVs, in which the binding sites for E-box and/or Ikaros in the proximal LMP1 promoter were mutated independently or simultaneously. As reported, a short mutation of the *cis*-acting element (-67 to -55, mEbox/Ikaros) increased LMP1 expression and even the transformation efficiency of primary B cells. Unexpectedly, however, mutation of the E-box or Ikaros motif had little effect on LMP1 expression, suggesting that an as yet undiscovered transcriptional repressor might bind to the sequence. Alternatively, either E-box or Ikaros may be sufficient for suppression of LMP1 expression.

## Materials and Methods

### Cells and Reagents

HEK293 and HEK293EBV-bacterial artificial chromosome (BAC) cells were maintained in Dulbecco’s modified Eagle’s medium (Sigma) supplemented with 10% fetal bovine serum. Akata(-), AGS-EBV, AGS and LCL cell lines were cultured in RPMI-1640 medium supplemented with 10% fetal bovine serum. Antibodies against LMP1, EBNA1 and tubulin have been described previously (Murata et al., 2016). Horseradish peroxidase-conjugated goat antibodies to mouse/rabbit IgG were obtained from Amersham Biosciences. pLMP1/ED-L1-FLuc and pcDNABZLF1 were described previously (Noda et al., 2011). The control renilla luciferase vector pRL-null was purchased from Promega. pLMP1/ED-L1(mEbox/Ikaros)-FLuc was produced by site-directed mutagenesis using the following primers: TCTTACATCGCGTTACTCTGACGTAGCC and TTCCGGTAGGCCCGGGGGGATTTGCGG. To create single mutations in the E-box site and the Ikaros site, the following primers were used: CATGTTACTCTGACGTAGCCG and AGTTTCTTGGGATGTAGGCCC for pLMP1/ED-L1(mEbox)-FLuc, GGTAAGAAACACGCGTTACTCTG and ATGTAGGCCCGGGGGGATT for pLMP1/ED-L1(mIkaros)-FLuc. These mutations were also introduced into EBV-BAC as described below.

### Genetic Manipulation of EBV-BAC DNA and Cloning of HEK293 Cells

EBV-BAC DNA was a kind gift from W. Hammerschmidt (Delecluse et al., 1998). Homologous recombination was carried out in *E. coli* as described previously (Murata et al., 2009).

The intermediate, EBV-BAC LMP1 Neo/st, was prepared previously (Noda et al., 2011). The Neo/st cassette in the intermediate DNA was then replaced using the next transfer vector DNA, which contained mutations in the E-box and/or Ikaros motifs of the LMP1 promoter. For mutation of both motifs, the CACGCG motif of the E-box site and the TGGGAT motif of the Ikaros site (bold) (TAC**ATCCCA**AGAAA**CACGCG**TT) were mutated to give the following sequence: TAC**CGGAAT**CTTAC**ATCGCG**TT, as described in a previous report (Sjöblom-Hallén et al., 1999). The sequences of the single mutations at the E-box sites were TAC**ATCCCA**AGAAA**CTCATG**TT. The single Ikaros mutation had the sequence TAC**ATGGTA**AGAAA**CACGCG**TT.

Electroporation of *E. coli* was performed using the Gene Pulser III (Bio-Rad). NucleoBond Bac100 (Macherey-Nagel) was used for purification of EBV-BAC DNA. Recombination was confirmed by amplifying the promoter region by PCR, followed by electrophoresis of the *Bam*HI- or *Eco*RI-digested viral genome and sequencing analysis. HEK293 cells were transfected with EBV-BAC DNA by using Lipofectamine 2000 reagent (Invitrogen) and cultured on 10 cm dishes in the presence of 150 μg/ml hygromycin B for 10–15 days to clone GFP-positive cell colonies as described previously (Murata et al., 2009). At least 10 hygromycin-resistant, GFP-positive clones were isolated for each recombinant virus and two or three typical clones were used in the following analyses.

### Transfection, Luciferase Assays, and Immunoblotting

Luciferase assays and immunoblotting were carried out as described previously (Noda et al., 2011). Briefly, for luciferase assay, AGS or AGS-EBV cells were transfected with a firefly luciferase vector, in which the luciferase gene was driven by the wild-type (WT) (pLMP1/ED-L1-FLuc) or mutated LMP1 promoter using Lipofectamine 2000 (Invitrogen). To normalize transfection efficiency, the control renilla luciferase vector pRL-null was cotransfected. After 1 day, cell lysates were subjected to luciferase assay using the Dual-Luciferase Reporter Assay System (Promega). For immunoblotting, cells were lysed in sample buffer containing SDS and 2-mercaptoethanol, and subjected to SDS-PAGE followed by protein transfer to PVDF membranes. After blocking with skim milk, the membranes were incubated with primary, followed by secondary, antibodies, and the signals were detected via chemiluminescence.

### Quantitative Real-Time RT-PCR (qRT-PCR)

The TriPure Isolation Reagent (Roche) was used for purification of total RNA. After isopropanol precipitation and ethanol washing, total RNA pellets were dissolved in nuclease free water (Promega). Reverse transcription and real-time PCR (RT-PCR) reactions were carried out using the One-Step SYBR PrimeScript RT-PCR Kit II (TaKaRa) and Real-Time PCR System 7300, as described previously (Murata et al., 2016). The primers used for detection of the LMP1 gene were as follows; 5′-CTATTCCTTTGCTCTCATGC-3′ and 5′-TGAGCAGGAGGGTGATCATC-3′.

### Quantitative Real-Time PCR (qPCR) of EBV DNA

We normalized LMP1 mRNA levels with relative viral DNA copy number because viral copy number can affect the expression levels. The protocol of quantification and primers have been described previously (Yoshida et al., 2017). Briefly, cells were harvested from the aliquot of the qRT-PCR sample, and subjected to protease K treatment and DNA extraction, followed by determination of viral DNA levels by qPCR using FastStart Universal Probe Master (Roche Applied Science).

### B Cell Transformation Assay

Wild-type or mutant EBVs were obtained from the HEK293EBV-BAC cell culture supernatants. To determine viral titers, Akata(-) cells were infected with the supernatants and, after 2 days, the proportions of GFP-positive cells were counted using flow cytometry (FACSCalibur, Becton Dickinson). By reference to these percentages of EGFP-positive cells, the viral titers were normalized by adding control medium. Peripheral blood mononuclear cells (PBMCs) were collected from healthy adult donors who provided written informed consent, according to protocols approved by the Institutional Review Board of Nagoya University. PBMCs were seeded into 96-well plates at 1 × 10^5^ cells/well, and then infected with serial dilutions of adjusted WT or mutant virus suspensions. Infected cells were cultured with cyclosporin A. After 3 weeks, the 50% transforming doses were calculated.

## Results

### The Short Mutation in the LMP1 Promoter Increased Promoter Activity

To search for the negative regulator(s) of LMP1 expression, we here focused on a short nucleotide sequence in the proximal LMP1 promoter (-67 to -55), which was reported to function as negative *cis*-acting element (Sjöblom-Hallén et al., 1999). It was later confirmed that this region contains a binding motif for E-box transcription factors, and subsequently, it was suggested that this sequence also contained a recognition motif for the Ikaros transcription factor (Jansson et al., 2007).

**FIGURE 1:**
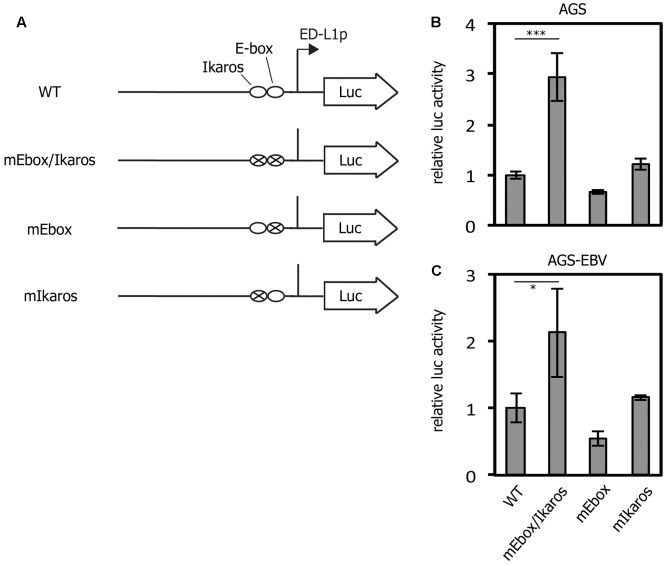
Reporter assays confirmed the importance of the E-box/Ikaros motif for LMP1 promoter activation. **(A)** Diagram of pLMP1/ED-L1-FLuc and its derivatives. The firefly luciferase gene is driven by the proximal wild-type LMP1 promoter (WT). The pLMP1/ED-L1(mEbox/Ikaros)-FLuc (mEbox/Ikaros), pLMP1/ED-L1(mEbox)-FLuc (mEbox), or pLMP1/ED-L1(mIkaros)-FLuc (mIkaros) plasmids carry the indicated mutations. **(B,C)** Results of the luciferase assays. AGS **(B)** and AGS-EBV **(C)** cells were transfected with pLMP1/ED-L1-FLuc (WT), pLMP1/ED-L1(mEbox/Ikaros)-FLuc (mEbox/Ikaros), pLMP1/ED-L1(mEbox)-FLuc (mEbox), or pLMP1/ED-L1(mIkaros)-FLuc (mIkaros), together with the pRL-null control vector. After 24 h, cells were lysed and subjected to luciferase assays. Relative firefly luciferase activity of mEbox after normalization to Renilla luciferase is shown as the fold change in activation over that of the WT. Each bar represents the mean and standard deviation of three independent transfections. Student’s *t*-test was performed. ^∗^*p* < 0.05 and ^∗∗∗^*p* < 0.005.

To evaluate the importance of these motifs, we first carried out luciferase assays (**Figure 1**). pLMP1/ED-L1-FLuc was generated by inserting the proximal LMP1 promoter sequence into pGL4.10 (Promega). Then, the E-box and/or the Ikaros motifs were mutated by site-directed mutagenesis, separately or together (**Figure 1A**). Identical quantities of these vectors were transfected into AGS (**Figure 1B**) and AGS-EBV (**Figure 1C**) cells and, after 24 h, subjected to luciferase assays. Mutations in the short nucleotide sequence (-67 to -55) that contains the E-box and Ikaros sites (mEbox/Ikaros) caused a mild increase in promoter activity in EBV-negative (AGS) and -positive (AGS-EBV) cells (2.9- and 2.1-fold, respectively). Unexpectedly, mutation of only the E-box site did not activate, and indeed slightly decreased promoter activity (0.66- and 0.54-fold, respectively in AGS and AGS-EBV cells). The single Ikaros mutation also did not increase promoter activity, if any (1.2- and 1.2-fold, respectively).

### Construction of Recombinant EBV Mutated in the LMP1 Promoter

**FIGURE 2:**
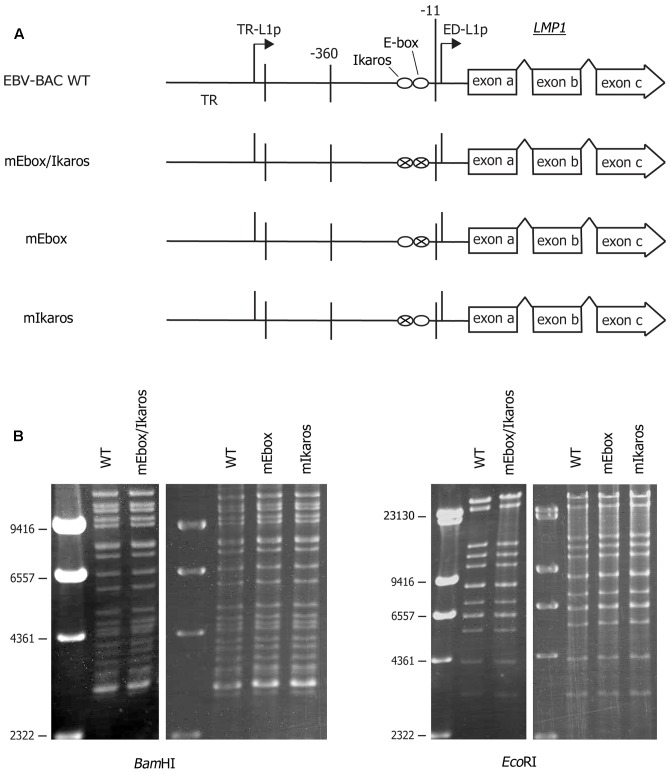
Construction of recombinant EBV featuring a mutation in the LMP1 promoter. **(A)** Schematic arrangement of recombination of the EBV genome using tandemly arranged neomycin-resistance and streptomycin-sensitivity genes (Neo/st). The B95-8 ED-L1 LMP1 promoter (–360 to –11) was first replaced with the Neo/st cassette, which was then replaced with mutated sequences (ringed X) to construct mutant EBV-BACs. TR, terminal repeat; TR-L1p, TR leftward promoter 1 (distal LMP1 promoter); ED-L1p, *Eco*RI-Dhet fragment leftward promoter 1 (proximal LMP1 promoter). **(B)** Electrophoresis of recombinant virus genomes. Recombinant EBV genomes were digested with *Bam*HI or *Eco*RI and resolved on an agarose gel.

To extend the reporter assay results, recombinant EBV carrying mutations in the E-box and/or Ikaros sites in the proximal ED-L1 LMP1 promoter was prepared (**Figure 2A**). A portion of the LMP1 ED-L1 promoter sequence (-360 to -11) containing the E-box site was first replaced with a marker cassette (Neo/st) to prepare the intermediate, which was then exchanged for the mutated sequence (marked as ‘×’ in **Figure 2A**) at the E-box and/or Ikaros motifs to prepare EBV-BAC mutants. Sequencing analysis confirmed that the EBV-BAC mutants contained the intended mutations. Electrophoresis of *Bam*HI- or *Eco*RI-digested BAC DNA confirmed the integrity and quality of the DNA (**Figure 2B**).

### LMP1 Expression in HEK293 Cells

**FIGURE 3:**
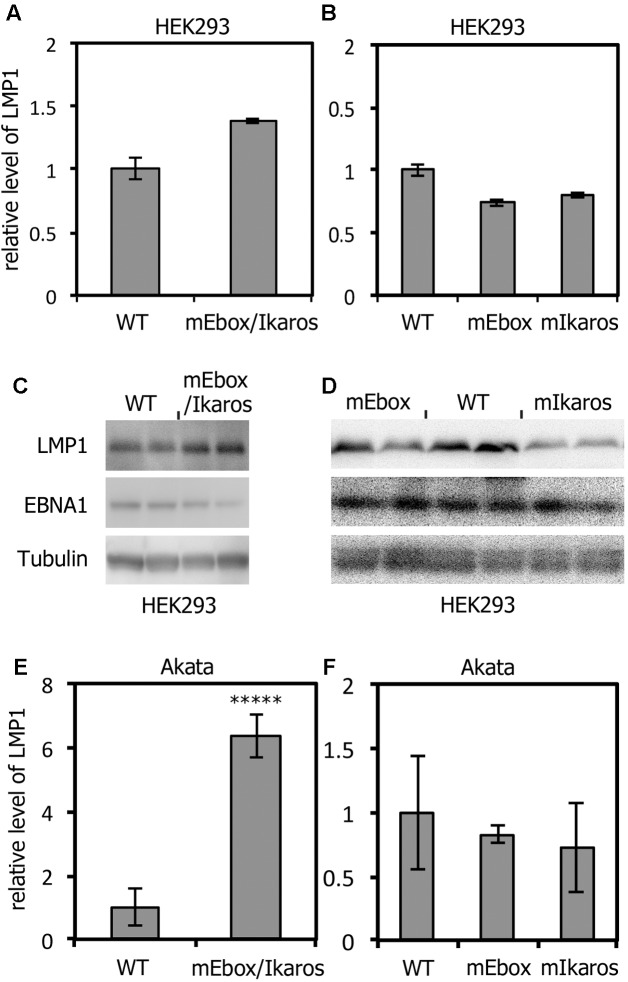
The mEbox/Ikaros mutation affected LMP1 expression in Akata cells but not in HEK293 cells. **(A–D)** EBV-BAC DNAs prepared as shown in **Figure 2** were introduced into HEK293 cells, and the LMP1 levels were measured by qRT-PCR **(A,B)** and immunoblotting **(C,D)** using anti-LMP1, -EBNA1, and -Tubulin antibodies. The relative levels of mRNA encoding LMP1 were normalized to the EBV copy numbers. **(E,F)** Infectious recombinant viruses (WT or mutants) were produced from the HEK293 cell lines described above, and equivalent numbers of viruses were used to infect EBV-negative Akata(-) cells. Cell RNA was harvested after 2 days and subjected to qRT-PCR to determine the levels of LMP1. Relative mRNA levels of LMP1 are shown after normalization to the EBV copy numbers. Each bar represents the mean and standard deviation of three independent infections. Student’s *t*-test was performed. ^∗∗∗∗∗^*p* < 0.002.

A virus-producing cell line, HEK293, was transfected with the recombinant EBV-BAC DNAs. Hygromycin-resistant, GFP-positive cell lines were cloned. In such cell lines, EBV is latently maintained as episomes. After preparing HEK293 cell clones harboring recombinant EBVs, the effect of the mutations on LMP1 expression was determined (**Figures 3A–D**). As EBNA2 is not detectable in HEK293EBV-BAC cells (Noda et al., 2011), the virus must express LMP1 in an EBNA2-independent manner. The levels of mRNA encoding LMP1 were almost identical in cells hosting either of the recombinant strains (**Figures 3A,B**). We next assessed the levels of LMP1 protein expressed by each of two typical independent clones of the WT and the mutants. LMP1 protein levels were similar among the WT and mEbox/Ikaros mutants (**Figure 3C**) and the single mutants (mEbox and mIkaros) (**Figure 3D**). LMP1 levels may be slightly higher in mEbox/Ikaros mutant cells (**Figures 3A,C**). The LMP1 protein levels in cells hosting the mIkaros virus (**Figure 3D**) were marginally reduced, for an unknown reason.

### LMP1 Expression Was Induced by the Mutation in Akata Cells

Following preparation of virus-producing HEK293 cell clones, the effect of mutations in the LMP1 promoter in B cells was determined. For this purpose, the Akata(-) cell line, which is an EBV-negative subclone derived from the EBV-positive Burkitt lymphoma cell line Akata was used. To prepare viral stock solutions, HEK293 cell clones harboring recombinant EBVs were transfected with a BZLF1 expression vector. Transfection of BZLF1 initiates lytic replication leading to production of viral progeny. Viral titers in the stock solutions were assayed using Akata(-) cells to adjust the numbers of infectious virus particles per milliliter. After adjustment, Akata(-) cells were inoculated with the viruses. LMP1 mRNA levels on day 2 were determined by qRT-PCR (**Figures 3E,F**). Expression of LMP1 increased 6.4-fold in cells infected with the short nucleotide (-67 to -55) mutant virus (**Figure 3E**, mEbox/Ikaros). In contrast, LMP1 levels were not significantly increased by the single mutations (**Figure 3F**, mEbox and mIkaros). Therefore, our results indicate that the short nucleotide sequence that spans both the E-box and Ikaros sites plays a suppressive role in terms of LMP1 transcription in the context of B cell infection, but binding of either of the E-box transcription factor or the Ikaros transcription factor alone does not explain the observed regulation.

### Increase in LMP1 Expression Induced by the Short Mutation in Primary B Cells

**FIGURE 4:**
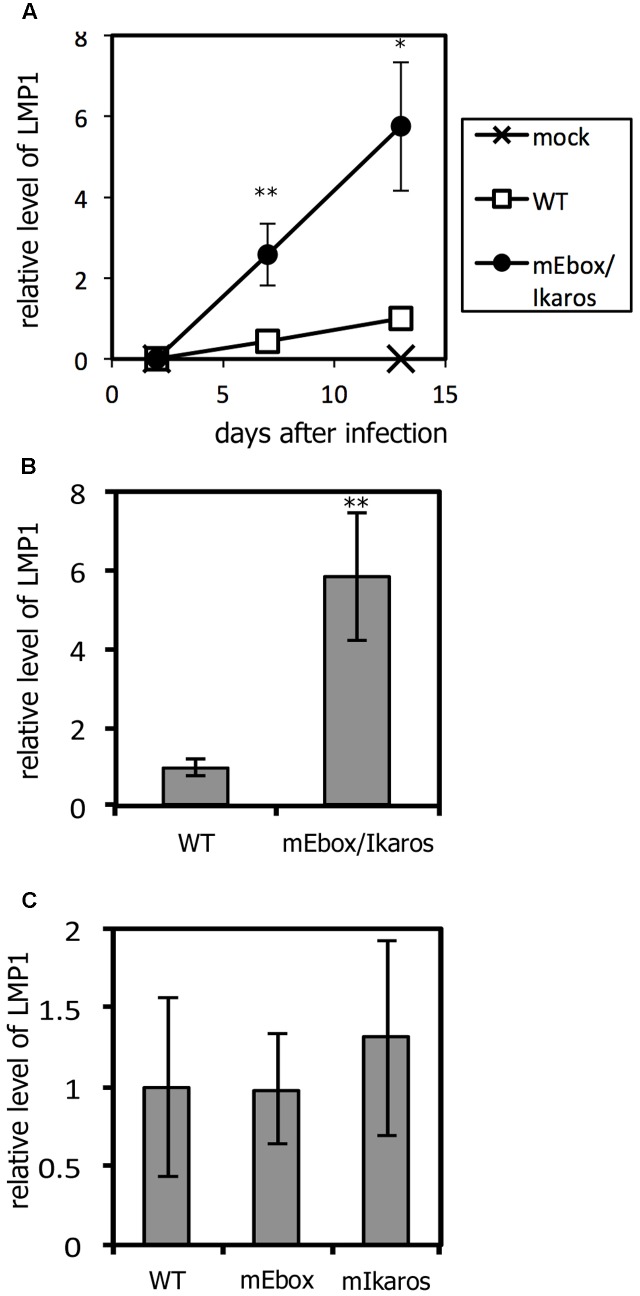
The mEbox/Ikaros mutation in the negative *cis*-element affected LMP1 expression in primary B cells. **(A)** LMP1 expression at early times after *de novo* infection. PBMCs (from donor A) were mock-infected or infected with EBV-BAC WT or mutant viruses. Cell RNA was collected at days 2, 7, and 13 after infection and subjected to qRT-PCR for LMP1 and LMP2 genes. Relative LMP1 mRNA levels after normalization to LMP2 levels are shown. The value of WT on day 13 was set as 1. **(B,C)** Likewise, PBMCs (from donors B and C, respectively) were infected with EBV-BAC WT or mutant viruses and harvested on day 14. The relative levels of mRNA encoding LMP1 were normalized to the EBV copy numbers. The mean and standard deviation of three measurements are plotted. Student’s *t*-test was performed. ^∗^*p* < 0.05 and ^∗∗^*p* < 0.02.

Because Akata cells are cancer cells, which are not the primary target of EBV in humans, the effects of mutations in primary B cells were next evaluated. To this end, the virus stock titer was normalized and used to infect PBMCs from a healthy donor. Cellular RNA was harvested on days 2, 7 and 13, and LMP1 expression levels were examined (**Figure 4A**). Two days after infection, LMP1 was undetectable in our assay in either the WT or the mutant. This result agrees with a previous paper to the effect that LMP1 transcription is markedly restricted for ∼1 week after infection of primary B cells (Price and Luftig, 2015). LMP1 expression in infected cells increased by day 13, and, importantly, transcription of LMP1 mRNA was notably higher (5.6-fold) in cells infected with the mEbox/Ikaros mutant on day 13 (**Figure 4A**). To explore the reproducibility, we repeated the experiment using primary PBMCs from another donor, and a different virus stock, and harvested viruses after 14 days. Compared to the WT gene, the LMP1 mRNA level was increased by 5.8-fold by the mEbox/Ikaros mutation (**Figure 4B**).

Recombinant EBVs with single mutations were also tested in **Figure 4C**. Expression of LMP1 was not increased by either the mEbox or mIkaros mutation.

**FIGURE 5:**
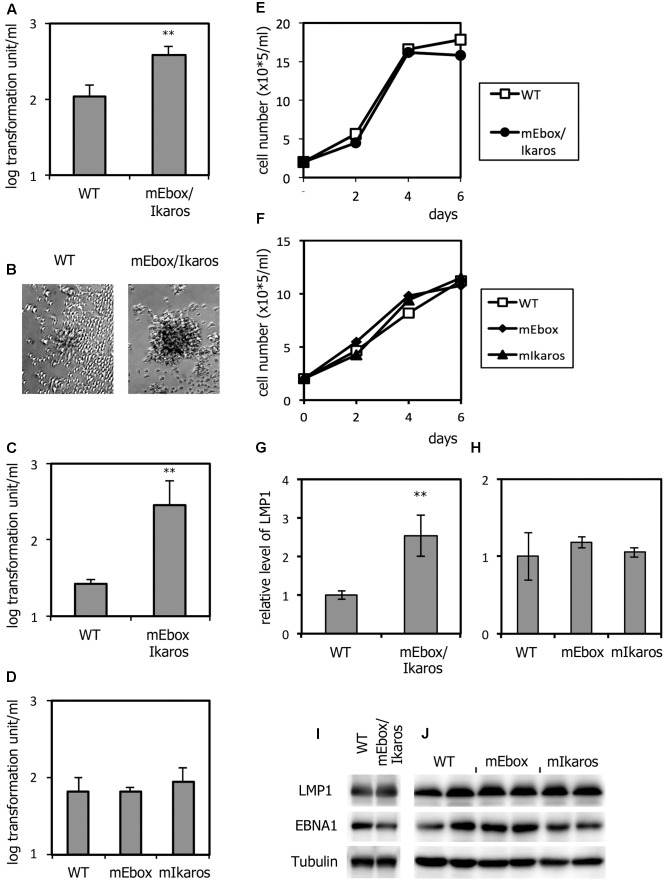
Transformation efficiency of recombinant EBVs carrying the mutations within the LMP1 promoter. **(A–D)** Viruses obtained from WT or mutant HEK293EBV-BACs were adjusted to 1 × 10^4^ GAU (green Akata unit) per milliliter, and infected with PBMCs in the presence of cyclosporine A. Three weeks later, transformation units per milliliter were determined. The mean and standard deviation of three independent assays are shown. Student’s *t*-test was performed. ^∗∗^*p* < 0.02. **(B)** Bright-field image of typical clumps of LCLs after 12 days. **(E,F)** Growth properties of LCLs. LCLs (2 × 10^5^ cells/ml) prepared in **(A,D)** were seeded and enumerated after 2, 4, and 6 days. **(G–J)** LCLs obtained in **(A,D)** were subjected to qRT-PCR **(G,H)** and immunoblotting **(I,J)**. The relative levels of mRNA encoding LMP1 were normalized to the EBV copy numbers. The means and standard deviations of three measurements are plotted. Student’s *t*-test was performed. ^∗∗^*p* < 0.02. PBMCs from donor A were used for **(A,B,E,I)**, those from donor B were used for **(C,G)**, those from donor B were used for **(D,F,H,J)**.

Next, whether the mutation in the LMP1 promoter influenced transformation efficiency was determined. PBMCs were infected with viruses after normalization, and cultured in the presence of cyclosporin A for 3 weeks. The immortalization efficiency of the mE-box/Ikaros mutant virus (3.8 × 10^2^/ml) was modestly but significantly higher than that of the WT (1.1 × 10^2^/ml) (**Figure 5A**). In addition, the clumps of LCLs infected with mE-box/Ikaros virus were markedly larger on day 12 than those infected with the WT virus (**Figure 5B**). Therefore, B cell transformation was significantly increased by the simultaneous mutation. We repeated the same experiment (**Figure 5C**) to confirm the robustness of the findings; we used primary PBMCs from another donor and a different virus stock. In this experiment, the transformation unit of the WT virus was 2.6 × 10^1^/ml and that of the mEbox/Ikaros mutant was higher at 2.9 × 10^2^/ml (**Figure 5C**). It is noteworthy that the effect of the mEbox/Ikaros mutation on LMP1 expression was more remarkable in the experiment shown in **Figure 5C**. The reason is not clear, but because the effect seems to depend on the donor, a host factor may be involved in this phenomenon. Consistently, the mEbox/Ikaros mutant exhibited higher transformation efficiency associated with larger cell clumps.

We then examined the effects of single mutations (mEbox or mIkaros). The transformation efficiency was not significantly altered by either of the mutations (mEbox or mIkaros) (**Figure 5D**).

Although LCLs infected with the short nucleotide (-67 to -55) mutant (mEbox/Ikaros) EBV grew more efficiently than the WT for at least for several weeks (**Figures 5A–C**), we observed that WT LCLs grew almost as efficiently as the mutant (mEbox/Ikaros, mEbox, and mIkaros) LCLs after ∼50 days (**Figures 5E,F**). The level of the LMP1 transcript in the mEbox/Ikaros LCLs, cultured for ∼50 days, was only slightly higher (2.5-fold) than that in the WT LCLs (**Figure 5G**). Such a low-level increase was not obvious upon immunoblotting (**Figure 5I**). It is speculated that LMP1 expression in the WT is increased or LMP1 transcription is silenced in the mEbox/Ikaros mutant, during prolonged cultivation. In addition, the LMP1 levels were similar among the LCLs of WT, mEbox, and mIkaros viruses (**Figures 5H,J**).

These results indicate that the short element in the proximal LMP1 promoter plays a role in suppression of LMP1 transcription for at least several weeks after infection of primary B cells.

## Discussion

In this study, the role of the short *cis*-acting element in the proximal LMP1 promoter (-67 to -55) was first evaluated by reporter assays (**Figure 1**). Mutation of the short sequence increased the promoter activity in both EBV-positive and -negative cells, as reported previously (Sjöblom-Hallén et al., 1999). The same mutation was then introduced into the EBV genome via bacterial recombination (**Figure 2**, mEbox/Ikaros). Mutation of the E-box and Ikaros motif not only induced LMP1 expression but also increased B cell transformation efficiency for at least several weeks (**Figures 3**–**5**).

Infection experiments such as these require precise determination and adjustment of viral titers. We assessed viral titers very carefully; after we determined titers using several dilution series, we calculated the volumes required equalize the titers of stocks used for infection or transformation experiments. We then re-evaluated the volume before actual infection of B cells. We are confident that the titers were precisely normalized because other viral genes, such as LMP2, were almost equally expressed when LMP1 level was increased by the mEbox/Ikaros mutation (**Figures 3**–**5**). Moreover, we present LMP1 transcript levels after normalization to EBV copy numbers, so that any difference in the multiplicity of infection between the clones can be ignored.

Because the short region contains binding motifs for the E-box and Ikaros transcription factors, we then mutated the motifs separately. Unexpectedly, mutation of the E-box or Ikaros motif had little or no positive effect on LMP1 expression, either in reporter assays (**Figure 1**) or during infection (**Figures 3**–**5**). These results suggest that the short sequence in the proximal LMP1 promoter retains a negative regulatory element, but the E-box and Ikaros transcription factors may not be the major players in terms of suppression. It is possible that an as yet unknown factor, which binds to the short region, may be involved in LMP1 suppression. Alternatively, either of the transcription factors might be sufficient for repression of LMP1.

It has been reported that LMP1 expression remains very low for several days after EBV infection of primary B cells and increases thereafter (Price and Luftig, 2015). As our present study, as well as a previous report (Sjöblom-Hallén et al., 1999), indicate that the short *cis*-acting element in the proximal LMP1 promoter is involved in suppression of LMP1, we speculated that this region suppresses LMP1 transcription immediately after primary infection of B cells. However, expression of LMP1 was not detectable 2 days after infection despite mutation in the region. Therefore, a further, as-yet-unknown, *cis*-acting motif may serve to inhibit LMP1 transcription upon infection of primary B cells.

LMP1 expression was higher in the mutant within the negative *cis*-acting element beginning 1 week after infection (**Figure 4A**) and lasted for weeks; this explains the higher immortalization efficiency and larger clumps (**Figures 5A–C**). However, the magnitude of the difference in cell growth speed decreased after 50 days (**Figure 5E**). It is thus possible that the LMP1 promoter activity of the mutant virus is silenced during prolonged cultivation. Alternatively, a subset of cells infected with WT EBV might develop higher LMP1 expression and thus acquire a growth advantage, resulting in their dominance over time. In fact, LMP1 expression during mEbox/Ikaros infection was about sixfold higher 14 days after infection (**Figure 4B**), decreasing to 2.5-fold after 50 days (**Figure 5G**).

Since cells infected with the virus mutated in the negative *cis*-acting element proliferate more rapidly than those infected with WT virus for at least several weeks, the reasons for the ubiquity of this *cis*-acting motif in WT EBV is unclear. One possibility is that excess LMP1 levels provoke a stronger immune reaction *in vivo*. It is also known that excess LMP1 expression is cytotoxic at least under certain conditions (Lu et al., 1996; Ito et al., 2014). Alternatively, this motif might benefit lytic replication while suppressing B cell immortalization. Interestingly, it was reported recently that LMP1 was not essential for proliferation of EBV-positive B cells in a mouse model if T cells from the same donor are available, possibly due to the supply of survival signals by T cells (Ma et al., 2015). Therefore, higher LMP1 expression might not necessarily imply greater proliferation of EBV-positive B cells *in vivo*.

In this study, we showed the downregulation of LMP1 transcription by a *cis*-acting element in the proximal LMP1 promoter. Interestingly, LMP1 is regulated at multiple levels apart from transcription. For example, EBV-encoded microRNAs reduce LMP1 expression (Lo et al., 2007; Ramakrishnan et al., 2011; Lin et al., 2015). LMP1 protein is rapidly degraded by the ubiquitin/proteasome-dependent pathway (Aviel et al., 2000). In addition, it has recently been shown that LMP1 can be efficiently incorporated into multivesicular bodies and exosomes in the manner dependent on CD63. Since knockdown or knockout of CD63 resulted in upregulation of signaling pathways, such as NFκB and MAPK, LMP1 function appears to be tightly regulated by the CD63-mediated endosomal/exosomal pathway (Verweij et al., 2011; Hurwitz et al., 2017). Such intricate control mechanisms suggest that LMP1 regulation is very sensitive, indeed “careful,” and that EBV has evolved elaborate regulatory mechanisms during a long history of co-existence with humans.

We here demonstrated a suppressive function of the short *cis*-acting motif within the proximal LMP1 promoter using reporter assays and recombinant viruses. Our results provide novel insight into the transcriptional regulation of LMP1, the major oncoprotein of EBV.

## Author Contributions

MY and TM designated the experiments. MY, TM, KA, YN, TW, and HM carried out experiments. YS, FG, HK, and TM supervised and discussed the experiments and data. MY, HK, and TM prepared the manuscript.

## Conflict of Interest Statement

The authors declare that the research was conducted in the absence of any commercial or financial relationships that could be construed as a potential conflict of interest.
